# Would Major Incompatible Blood Type Lung Transplants be Standard Care?

**DOI:** 10.3389/ti.2022.10666

**Published:** 2022-08-17

**Authors:** Akira Akabayashi, Eisuke Nakazawa

**Affiliations:** ^1^ Department of Biomedical Ethics, University of Tokyo Faculty of Medicine, Tokyo, Japan; ^2^ Division of Medical Ethics, New York University School of Medicine, New York, NY, United States

**Keywords:** ethics, donor, lung, ABO-incompatible, coercion

Dear Editors

On 12 April 2022, Kyoto University Hospital (Japan) announced a successful incompatible blood type lung transplant from living donors (1). Specifically, a right lower lobe from a father with blood type B and a left lower lobe from a mother with blood type O were transplanted into their pre-teen/teenage daughter, with blood type O, who had severe obstructive bronchiolitis. This **living-donor** lung transplant is noteworthy because it was performed in a **major incompatible state** (wherein a recipient with type O blood receives a transplant from a donor with type A/B/AB). To date, only one major incompatible blood type lung transplant (MIBTLT), from a brain-dead donor, has been reported in Germany (2). The patient from the German operation is alive as of 5 July 2022, without any signs of long-term chronic rejection (as per personal communication from Dr. Axel Haverich).

In this case, Rituximab was administered 3 weeks prior to surgery, immunosuppressive drugs were administered, and plasmapheresis was performed to remove anti-B antibodies. This is the same strategy used for kidney and liver incompatible blood type transplants. Unlike kidneys and livers, lungs are subject to strong rejection and are vulnerable to infection, due to their exposure to air. Therefore, lung transplantation from organs with incompatible blood types is considered difficult.

We will make seven points.

First, the immunological mechanism in the lungs: This method has not been successful in lungs, despite favorable outcomes having been achieved in livers and kidneys. However, it was successful when **living-donor** lungs were used. The originality of this case pertains to **living-donor** lungs being used for a **MIBTLT**.

Most candidate lungs are considered unsuitable because of lung injuries that occur with brain-death and ICU-related complications (i.e., barotrauma or lung edema associated with fluid resuscitation) (3). However, the success of this case infers that **MIBTLT** may be feasible in clinical settings if the underlying immunological mechanisms are clarified. We assume that different immunological conditions exist between the lungs of brain-dead versus living-donor.

The clinical preservation time limit for lungs seems to be roughly 8 h. It is important to note that the clinical preservation time was absent in this case. The success of the **MIBTLT** procedure may be due to the freshness of the living-donor lungs. We need to know how long after brain-death the lung was transplanted in the German operation. Studying what immunological and histological events occur in those 8 hours of clinical preservation is necessary.

We are fully aware of the Toronto group’s attempts at *ex-vivo* lung perfusion (**EVLP**). However, it appears that the main purpose of **EVLP** is to prolong clinical preservation time. **EVLP** trials have so far provided little insight into the immunological mechanisms that make **MIBTLT** possible.

Second, information of successful cases: The recipient who underwent **MIBTLT** from a brain-dead lung donor, in Germany in 2007 (2), has maintained a high QOL with no chronic rejection in the 15 years following the operation. The common belief surrounding transplants from a major incompatible blood type has been that “liver and kidney transplants can work (even with ABO major incompatibility), but lungs don’t work.” The prognosis and condition of the recipient urged us to share this information with the global transplant community immediately.

Third, potential to address emergencies: ABO **MIBTLT**, using living-donor lungs, may be a treatment option when no ABO-compatible donor is present—typically during emergency surgery. EVLP allows for transplantation with extended ischemic time, since the issue of ischemic time is not relevant to living-donor lungs.

Fourth, evidence for effectiveness of EVLP: Is EVLP effective for ABO **MIBTLT**? A retrospective study that examined data from the Toronto Lung Transplant Program database (N = 906) (4) reported that outcomes were not different amongst procedures where total preservation time exceeded 12 h versus those where total preservation time was less than 12 h. However, the study in question did not list ABO incompatibility as being compatible, minor incompatible, or major incompatible. It is unclear whether all surgeries from the Toronto Lung Transplant Program were compatible. Comparing the outcomes of **MIBTLT** with those of compatible and minor incompatible lung transplantations is essential. If the outcome of the former is significantly worse than the others, **MIBTLT** should be considered a compassionate treatment rather than a standard care.

Fifth, cost: The **EVLP** technique requires a circuit with multiple, complex components, depending on the device used. It commonly includes some form of drainage from the left atrium, chamber reservoir, centrifugal pump, membrane gas/heat exchanger, filtered gas. It commonly includes some form of drainage from the left atrium, chamber reservoir, centrifugal pump, membrane gas/heat exchanger, filtered gas line for deoxygenation, leucocyte filter, inflow cannula into the pulmonary artery and a ventilator connected to the trachea (5) Additionally, **EVLP** takes 12 h to complete. Methods to reduce the cost of EVLP such as hubbing (6) are proposed; however, the expense of the equipment and time of medical personnel remain substantial.

It is unclear whether the use of Rituximab, immunosuppressants, and plasmapheresis is less expensive. A rigorous cost-benefit analysis must be done to determine which of these options is more inexpensive. While beneficiary’s financial burden may increase, we do not support treatments that could be characterized as those that “only the rich can afford.”

Sixth, ethical issues: Serious ethical issues emerge when considering **MIBTLT**—particularly regarding the donor selection process. In Japan, the number of brain-dead donors is remarkably small, while living-donor transplants are the norm (7). If **MIBTLT** becomes standard care, refusal to be a donor (based on having a different blood type) becomes difficult. The psychological pressure that is placed on potential donors (who are often family members) will undoubtedly increase. In the case of living-donor transplantation, voluntariness is essential, since donors are healthy individuals. Strict regulations need to be established in each country to prevent coercion of potential donors.

Lastly, this is the first case of ABO **MIBTLT** being performed in living-donor lungs, where a favorable outcome was achieved. This report is significant because the success of ABO **MIBTLT** (along with other attempts, such as **EVLP**) may increase treatment selection, thereby reducing potential organ shortages.

## References

[1] The Japan Times. In World First, Kyoto Hospital Completes Lung Transplant on Patients with Different Blood Types. The Jpn Times (2022). Apr 13.

[2] Strüber M Warnecke G Hafer C Goudeva L Fegbeutel C FiScher S Intentional ABO-Incompatible Lung Transplantation. Am J Transpl (2008) 8:2476–8. 10.1111/j.1600-6143.2008.02405.x 18808407

[3] Cypel M Keshavjee S. Extracorporeal Lung Perfusion (*Ex-vivo* Lung Perfusion). Curr Opin Organ Transpl (2016) 21(3):329–35. 10.1097/MOT.0000000000000320 27145198

[4] Yeung JC Krueger T Yasufuku K de Perrot M Pierre AF Waddell TK Outcomes after Transplantation of Lungs Preserved for More Than 12 H: A Retrospective Study. Lancet Respir Med (2017) 5(2):119–24. 10.1016/S2213-2600(16)30323-X 27866861

[5] Garijo JM Roscoe A. *Ex-vivo* Lung Perfusion. Curr Opin Anaesthesiol (2020) 33(1):50–4. 10.1097/ACO.0000000000000804 31688085

[6] Yeung JC Cypel M Keshavjee S. *Ex-vivo* Lung Perfusion: The Model for the Organ Reconditioning Hub. Curr Opin Organ Transplant (2017) 22(3):287–9. 10.1097/MOT.0000000000000404 28301389

[7] Akabayashi A Nakazawa E Ozeki-Hayashi R Tomiyama K Mori K Demme RA Twenty Years after Enactment of the Organ Transplant Law in Japan: Why Are There Still So Few Deceased Donors? Transpl Proc (2018) 50:1209–19. 10.1016/j.transproceed.2018.02.078 29880339

